# Radiomics based analysis to predict local control and survival in hepatocellular carcinoma patients treated with volumetric modulated arc therapy

**DOI:** 10.1186/s12885-017-3847-7

**Published:** 2017-12-06

**Authors:** Luca Cozzi, Nicola Dinapoli, Antonella Fogliata, Wei-Chung Hsu, Giacomo Reggiori, Francesca Lobefalo, Margarita Kirienko, Martina Sollini, Davide Franceschini, Tiziana Comito, Ciro Franzese, Marta Scorsetti, Po-Ming Wang

**Affiliations:** 1 Radiotherapy and Radiosurgery Department, Humanitas Clinical and Research Hospital, Rozzano, Italy; 2 Nuclear Medicine Department, Humanitas Clinical and Research Hospital, Rozzano, Italy; 3 Department of Biomedical Sciences Humanitas University, Rozzano, Italy; 4 Department of Radiation Oncology, Cheng-Ching General Hospital, Taichung, Taiwan; 5 Polo Scienze Oncologiche ed Ematologiche, Fondazione Policlinico, Universitario Agostino Gemelli, Rome, Italy; 6 Humanitas Cancer Center and Research Hospital, Via Manzoni 56, 20089 Rozzano, Milano, Italy

**Keywords:** Hepatocellular carcinoma, Liver cancer, VMAT, Radiomics; texture analysis, Outcome prediction

## Abstract

**Background:**

To appraise the ability of a radiomics based analysis to predict local response and overall survival for patients with hepatocellular carcinoma.

**Methods:**

A set of 138 consecutive patients (112 males and 26 females, median age 66 years) presented with Barcelona Clinic Liver Cancer (BCLC) stage A to C were retrospectively studied. For a subset of these patients (106) complete information about treatment outcome, namely local control, was available. Radiomic features were computed for the clinical target volume. A total of 35 features were extracted and analyzed. Univariate analysis was used to identify clinical and radiomics significant features. Multivariate models by Cox-regression hazards model were built for local control and survival outcome. Models were evaluated by area under the curve (AUC) of receiver operating characteristic (ROC) curve. For the LC analysis, two models selecting two groups of uncorrelated features were analyzes while one single model was built for the OS analysis.

**Results:**

The univariate analysis lead to the identification of 15 significant radiomics features but the analysis of cross correlation showed several cross related covariates. The un-correlated variables were used to build two separate models; both resulted into a single significant radiomic covariate: model-1: *energy p* < 0.05, AUC of ROC 0.6659, C.I.: 0.5585–0.7732; model-2: *GLNU* *p* < 0.05, AUC 0.6396, C.I.:0.5266–0.7526.

The univariate analysis for covariates significant with respect to local control resulted in 9 clinical and 13 radiomics features with multiple and complex cross-correlations. After elastic net regularization, the most significant covariates were compacity and BCLC stage, with only compacity significant to Cox model fitting (Cox model likelihood ratio test *p* < 0.0001, *compacity p* < 0.00001; AUC of the model is 0.8014 (C.I. = 0.7232–0.8797)).

**Conclusion:**

A robust radiomic signature, made by one single feature was finally identified. A validation phases, based on independent set of patients is scheduled to be performed to confirm the results.

## Background

Hepatocellular carcinoma (HCC) is the third cause of cancer death and one of the most challenging oncological problems [1]. Surgery, although providing survival rates up to 70% at 5 years [2], is viable in a small fraction of patients (less than 1/3) because of advanced stage at diagnosis. In this clinical setting the use of radiotherapy was limited by severe radiation induced liver disease (RILD) [3–7]. After the introduction of intensity modulated radiotherapy (IMRT) and Volumetric modulated Arc Therapy (VMAT), a new hope emerged for radiotherapy in HCC patients [8–10]. Preliminary valuable data resulting from the use of VMAT also in association with stereotactic body radiation therapy (SBRT), were proved [11–14]. In this context, it would be important to develop and validated tools capable to predict for individual patients, the likelihood of tumor control and possibly of survival in order to better personalize the treatment offering. Textural analysis of diagnostic images is a very broad area of research which might lead to the definition of such tools. In particular, radiomics is an emerging field that converts imaging data into a high dimensional mineable feature space using a large number of automatically extracted data-characterization algorithms [15, 16]. Radiomics has being evaluated, in oncology, also as a potential prognostic indicator, useful for classifying patients and evaluating their assignment to risk categories in order to customize and tailor the prescribed oncological treatments [17–19]. While several investigations has been published on the use of radiomics in many cancer models [20–22] and the correlation between radiomics signatures to radiation treatment outcome, little is available for liver cancer.

In general, some studies were published concerning the use of texture analysis in the liver (primary hepatocellular carcinoma or metastatic disease) to either classify the lesion type or to facilitate the therapeutic decision. Echegaray [23] investigated (retrospectively on 29 patients with HCC) the possibility to identify robust radiomics features in CT image datasets, insensitive to segmentation processes and identified them in the intensity and texture families. The study was done testing multiple manual contouring by different radiologists and identifying automatic “core sample” regions of interest for the textural analysis. Chen [24] analyzed the prognostic value of texture features for hepatocellular carcinoma on a cohort of 61 patients who underwent hepatectomy. CT textural characteristics allowed to identify higher order features with potential prognostic value outperforming the more traditional predictors like the Barcelona Clinic Liver Cancer (BCLC) stage. Li [25] explored the potential of CT textural analysis to stratify patient with HCC and to help in the determination of the optimal therapeutic procedure among resection or arterial chemoembolization. Authors claimed that wavelet decomposition allowed a successful stratification of the patients although further validation was required. Raman [26] used radiomics analysis of CT data to classify different liver lesions types, with specific regard to hyper-vascularization. The predictive model they trained and validated (on a retrospective cohort) allowed to correctly classify adenomas, focal nodular hyperplasia and hepatocellular carcinoma with accuracy in the range of 91–99% while human observers had a correspondent accuracy in the range of 66–72%. Lubner [27] appraised the role of radiomics analysis of CT images for hepatic metastatic colorectal cancer, finding that primarily histogram based features were significantly associated to tumor grade in untreated liver metastases suggesting that two-dimensional (2D) texture analysis on single slices might be adequate. Similarly, Simpson [28] studied the correlation between texture analysis of CT datasets versus the risk of hepatic recurrence after resection of liver metastases in colorectal cancer patients. The hypothesis was that radiomics features could be predict the risk of future recurrence. The results confirmed that quantitative imaging features of the future liver remnant (after first resection) were predictive of hepatic disease-free survival (as well as of overall survival).

Literature search with various combinations of keywords like “radiomics” or “texture analysis” (and variants) in relation to “liver” and “radiotherapy” (and variants) did not provide any, suggesting that no published data might exist on the role of radiomics in the assessment and prediction of radiation treatment outcome for HCC patients.

In this study we present the results of a feasibility investigation aiming to identify possible radiomics signature applied to HCC patients for detecting a prognostic classification of such patients. Endpoints for the study were overall survival and the local control of the tumor after radiation treatment administered with volumetric modulated arc therapy.

## Methods

### Patients and treatment

Hundred thirty-eight consecutive HCC patients presented BCLC stage A to C and were eligible for curative or palliative radiotherapy and treated with VMAT as previously detailed in the retrospective analysis [29, 30]. All selected patients in the original retrospective study were either inoperable or not eligible for trans-arterial chemo embolization (TACE) treatments and received radiotherapy as primary treatment. In brief, patients with BCLC stages A to C, Child-Pugh stages A-B with single lesions larger than 5 cm or multi-nodular lesions larger than 3 cm were considered as eligible for radiotherapy. Portal vein thrombosis was present in about 53.6% of the cases. Dose prescription ranged from 45 Gy to 66 Gy depending upon stage, location of target and its size and general conditions of patient. All patients were treated with volumetric modulated arc therapy.

All patients were included in this new retrospective analysis and two cohorts (full or restricted) were defined according to the availability of survival data (available for all patients) and of objective response (for local control, available in a subset of patients).

All patients were treated between February 2009 and December 2010 according to the Helsinki declaration; ethical approval for retrospective analysis of data was provided by the institutional ethical review board.

Clinical evaluation was performed, with reference to baseline conditions: basic treatment outcome was measured in terms of in-field local control (visits included laboratory assessment and CT and MRI imaging (at 2 to 3 month intervals for at least 2 years and at 6 month intervals thereafter)) and patient overall survival and it was scored continuously with a median follow-up of 9 months (minimum 1 month, maximum 28 months). Tumor response was assessed using Response Evaluation Criteria in Solid Tumors (RECISTs) criteria. Local in field recurrence was defined by new enhancement or progressive disease with CT or MRI imaging during follow-up.

### Radiomics image analysis

The entire dataset of the treatment planning non-contrast enhanced CT images, all acquired with 3 mm slice thickness with an in-plane resolution of 0.8 mm, was analyzed to extract a number of textural features from the clinical target volumes contoured for the radiotherapy plans. The volumes subject to the textural analysis were defined as the clinical target volumes (CTV) manually contoured for the radiation treatment. The feature extraction was performed by means of the LifeX package [31, 32]. A total of 35 features were extracted from the analysis of the volumes inspected. These indices included conventional parameters, shape and size features, histogram-based features, second and high order-based features. The gray-level co-occurrence matrix (GLCM) [33]; the neighborhood gray-level different matrix (NGLDM) [34]; the grey level run length matrix GLRLM) [35] and the grey level zone length matrix (GLZLM) [36] were computed for each patient. The list of the corresponding features is provided in Table 1 while a detailed description of all the features, can be found in [37].

**Table 1:** Summary of the textural features used for the analysis

Feature name	Symbol/abbreviation
Geometry based and histogram based features	
Sphericity	–
Compacity	–
Skewness	–
Kurtosis	–
Entropy	Entropy_H
Energy	Energy_H
Gray-level co-occurrence matrix (GLCM)	
Homogeneity	–
Energy	–
Contrast	–
Correlation	–
Entropy	–
Dissimilarity	–
Neighborhood gray-level different matrix (NGLDM)	
Contrast	
Coarness	
Grey level run length matrix GLRLM)	
Short-Run Emphasis	SRE
Long-Run Emphasis	LER
Low Gray-level Run Emphasis	LGRE
High Gray-level Run Emphasis	HGRE
Short-Run Low Gray-level Emphasis	SRLGE
Short-Run High Gray-level Emphasis	SRHGE
Long-Run Low Gray-level Emphasis	LRLGE
Long-Run High Gray-level Emphasis	LRHGE
Gray-Level Non-Uniformity for run	GLNU
Run Length Non-Uniformity	RLNU
Run Percentage	RP
Grey level zone length matrix (GLZLM)	
Short-Zone Emphasis	SZE
Long-Zone Emphasis	LZE
Low Gray-level Zone Emphasis	LGZE
High Gray-level Zone Emphasis	HGZE
Short-Zone Low Gray-level Emphasis	LZLGE
Short-Zone High Gray-level Emphasis	LZHGE
Long-Zone Low Gray-level Emphasis	LZLGE
Long-Zone High Gray-level Emphasis	LZHGE
Gray-Level Non-Uniformity for zone	GLNU
Zone Length Non-Uniformity Zone Percentage	ZP

In addition to these groups, other parameters were derived for each volume: the sphericity and the compacity which measure the characteristics of the shape of the volume relatively to its regularity and compactness. From the histogram of the gray level distribution in the volume, a set of further parameters was obtained: the skewness (measure of the asymmetry of the distribution), the kurtosis (measuring weather the distribution is peaked or flat relative to a normal distribution), the entropy (randomness of the distribution) and the energy (uniformity of the distribution).

### Statistical analysis

Statistical analysis was performed using the open source R platform [38]. Univariate analysis was addressed to all clinical covariates (derived from the earlier retrospective analysis [29, 30] and defined as age, sex, portal vein thrombosis, tumor location, AJCC stage, BCLC stage, Okuda stage, Child-Pugh stage, previous Hepatitis, initial alpha-feto protein level, total radiotherapy treatment dose) and radiomics features in order to identify the most relevant predictors for clinical response using Pearson’s correlation test. Afterwards, for each radiomics covariate a procedure for detecting the threshold that better splits the different patient’s populations (responders and not-responders) was set up by dividing the population into group with a continuously moving covariate value in the range of all available values. The best threshold was defined as the value that obtains the lowest *p* value in the Pearson’s correlation test. A similar procedure has been set for the survival endpoint by using log-rank test p value in the Kaplan Meier statistic. The lowest p value corresponds even in this case to the best threshold separating populations. The mutual correlation between features was evaluated for the best performing covariate (*p* ≤ 0.05), in order to assess potential results redundancy. Covariates showing Pearson correlation test *p* ≥ 0.05 were considered not cross-related and used for multivariate analysis. Multivariate analysis was performed by logistic regression with backward elimination of not significant covariates for clinical response and by Cox-regression hazards model for survival. Models were evaluated by area under the curve (AUC) of receiver operating characteristic (ROC) curve. The standard ROC curve was computed by testing the sensitivity and specificity of the models in predicting the outcome from the selected predictors from the model. Calibration was evaluated with Hosmer and Lemen show goodness of fit test, *p* > 0.05 are accounted of not significant deviance from the theoretical perfect calibration. Missing value were dealt omitting cases not having all the variables available for analysis. In the survival analysis, the selection of covariates was obtained by elastic net regularization process in order to deal with multiple cross related covariates and reduce the risk of overfitting of the data. The elastic net regularization was introduced by Zou and Hastie [39] and aimed to improve both the accuracy of the prediction and the interpretation of the models. Elastic net regularization does automatic variable selection and continuous shrinkage, and can select groups of correlated variables allowing to identify the best predictors when a set of predictors is much more larger than the number of cases. Overall survival analysis was performed on the unrestricted dataset and local control on the restricted dataset.

## Results

A total number of 138 patients were enrolled in the study (*full dataset* - FD). Patients characteristics are summarized in Table 2. For all of them survival was available, but objective response (to determine local control) was only evaluated in a subset of cases (106, *restricted dataset* - RD). The analysis of overall survival showed a median OS of 10.1 months, with a median follow up time of 16.6 months.

**Table 2:** Demographic and clinical characteristics of the cohort of patients (full dataset)

Sex	Female: 26 (18.4%)
	Male: 112 (79.4%)
Age [years]	Mean: 64
	Median: 66
	St.dev: 11
	Range: 30–87
Portal Vein Thrombosis	No: 64 (46.4%)
	Yes: 74 (53.6%)
Tumour location	Right lobe: 57 (41.3%)
	Left lobe: 10 (7.2%)
	Bilateral: 71 (51.4%)
Stage T	T1: 8 (5.8%)
	T2: 10 (7.2%)
	T3: 120 (86.9%)
Stage N	N0: 114 (82.6%)
	N1: 24 (17.4%)
Stage M	M0: 116 (84.1%)
	M1: 22 (15.9%)
AJCC Stage	I: 7 (5.1%)
	II: 9 (6.5%)
	III: 83 (60.1%)
	IV: 39 (28.3%)
Okuda Stage	I: 31 (22.4%)
	II: 109 (77.6%)
BCLC Stage	A: 9 (6.5%)
	B: 29 (21.0%)
	C: 100 (72.5%)
Child-Pugh Stage	A: 96 (69.6%)
	B: 42 (30.4%)
Hepatitis (B/C)	No: 19 (13.8%)
	Yes: 119 (86.2%)
Initial Alpha-feto protein (ng/mL)	Mean: 11481
	Range: 2.4, >58300
Dose prescription	54Gy: 16 (11.6%)
	60Gy: 114 (82.6%)
	66Gy: 8 (5.8%)

Values refer to number of patients, % are relative to the total number of 138 patients

### Objective response (LC) analysis

Univariate analysis over clinical response versus radiomics features in FD using mobile threshold showed significant *p* values for *skewness* (threshold 6.87, *p* < 0.05), *contrast* (threshold 40.22, *p* < 0.01), and *dissimilarity* (threshold 4.37, *p* < 0.01); only the latter was used returning a better correlation with the outcome. Multivariate analysis with backward elimination, using the full range of covariates values didn’t return any significant result. Using the thresholding of covariates and dealing them as factors led to obtain a logistic multivariate model with only *contrast* as significant covariate (*p* < 0.05), the AUC of ROC of the model was 0.6649 (C.I. 0.5693–0.7605).

**Fig. 1:**
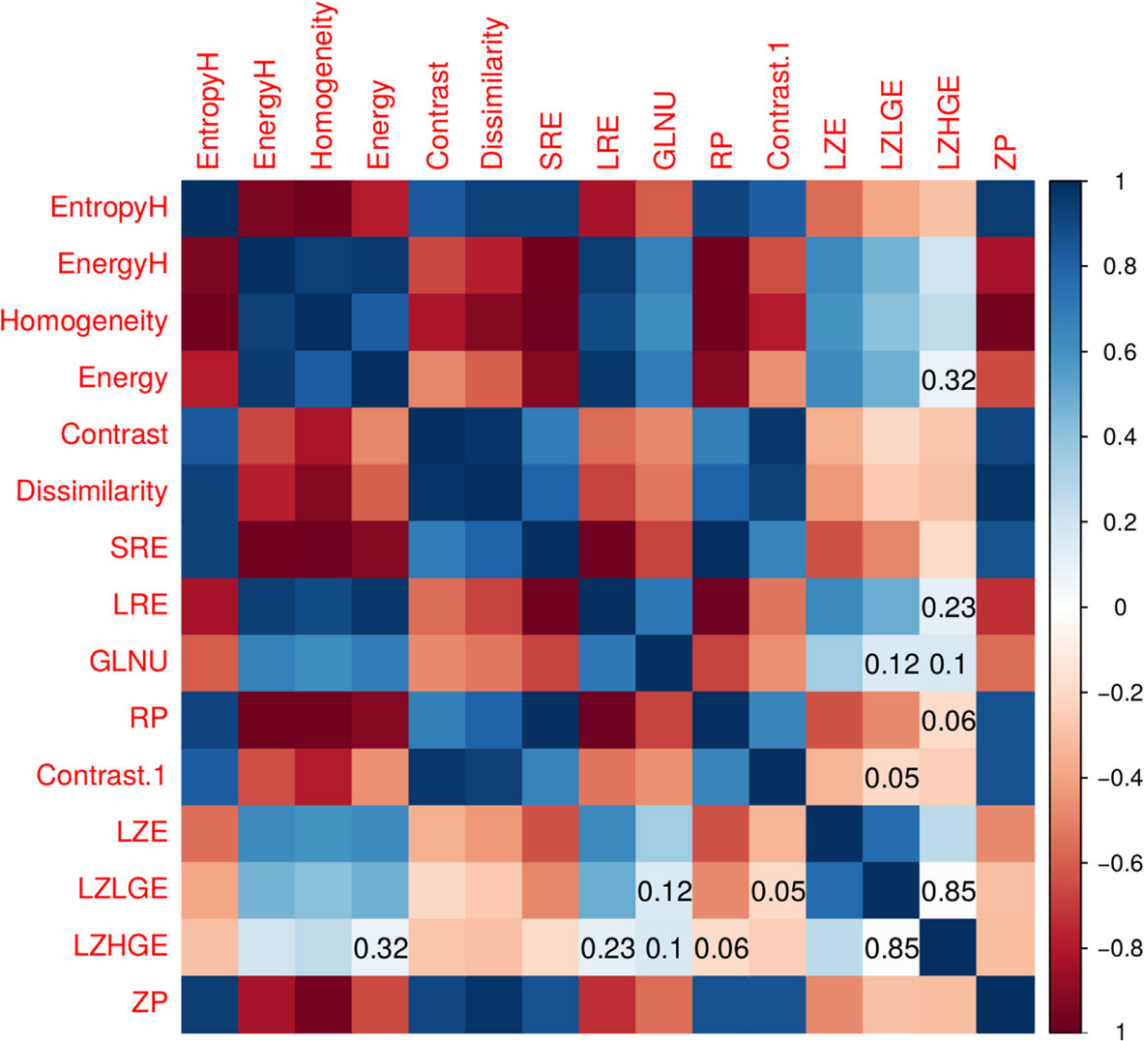
Cross correlation matrix. Numerical values correspond to Person correlation coefficient, achieved with Person correlation test *P*-Value >0.05 (low cross correlation)

The univariate analysis of RD showed several significant covariates. Results are summarized in Table 3. Analysis of cross correlation (Fig. 1) showed several cross related covariates, so only covariates with Pearson’s correlation test *p* > 0.05 were used for multivariate analysis in two different logistic models (Table 4) selecting two different groups of uncorrelated features. Both models result showed a single significant radiomics covariate (model 1: *energy* *p* < 0.05, AUC of ROC 0.6659, C.I. = 0.5585–0.7732; model 2: *GLNU* p < 0.05, AUC of ROC 0.6396, C.I. = 0.5266–0.7526).

**Table 3:** Univariate analysis in restricted dataset

Covariate	*P*-Value
Histogram based	
Entropy	0.03
Energy	0.03
Gray scale co-occurrence matrix (GLCM)	
Homogeneity	0.03
Energy	0.02
Contrast	0.03
Dissimilarity	0.04
Gray level run length matrix (GLRLM)	
SRE	0.02
LRE	0.02
GLNU	0.02
RP	0.02
Gray level zone length matrix (GLZLM)	
Contrast	0.04
LZE	0.03
LZLGE	0.01
LZHGE	0.03
ZP	0.03

*P*-values are the results of Mann-Whitney test

**Table 4:** Models built with not cross related covariates in the restricted dataset

LZHGE (model 1)	LZLGE (model 2)
Energy	GLNU
LRE	Contrast
GLNU	LZHGE
RP	–
LZLGE	–

### Survival data analysis

**Fig. 2:**
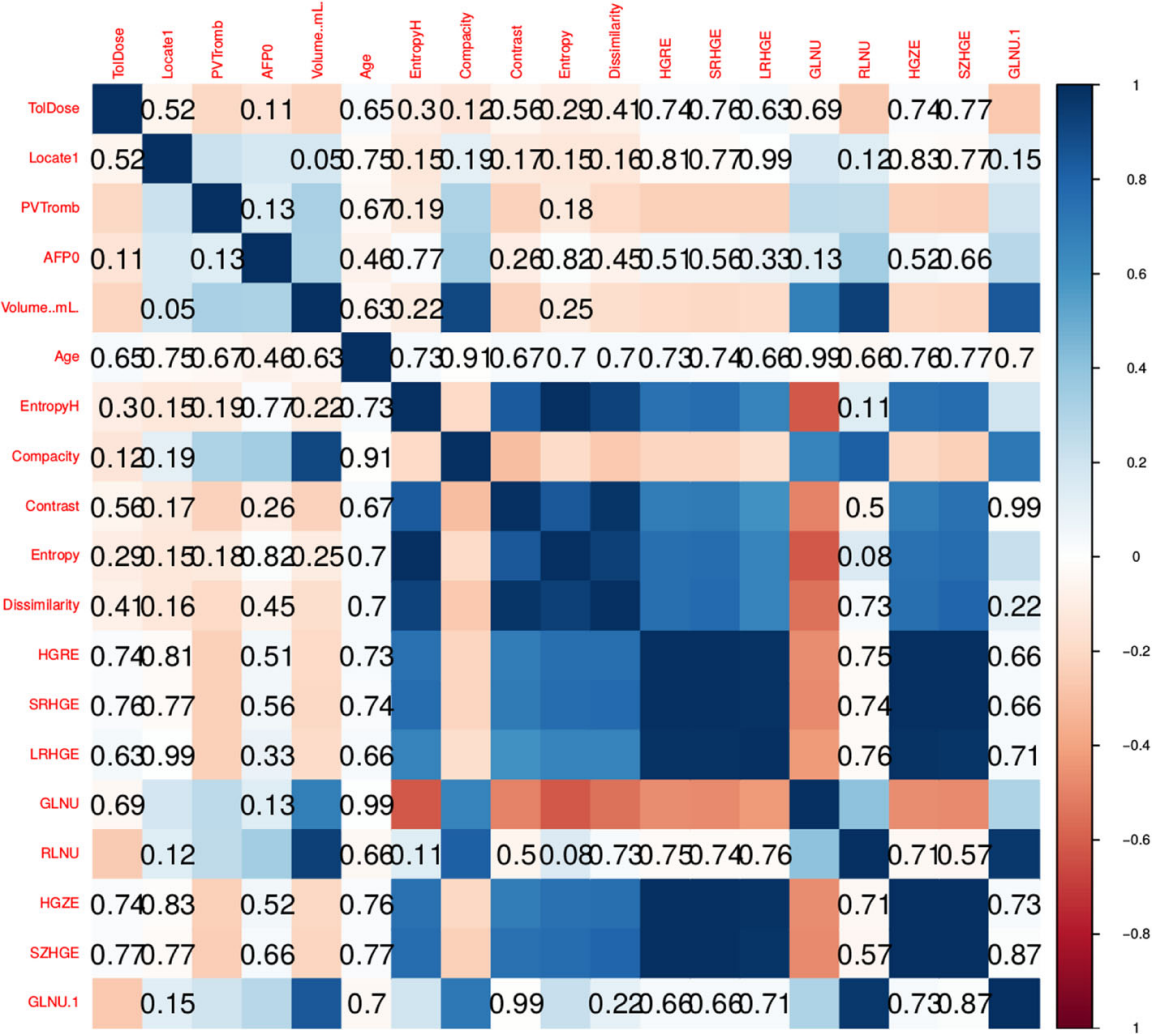
Cross correlation matrix for covariates used for log-rank test. Uncorrelated covariates are shown with Pearson correlation test *P*-Value

**Fig. 3:**
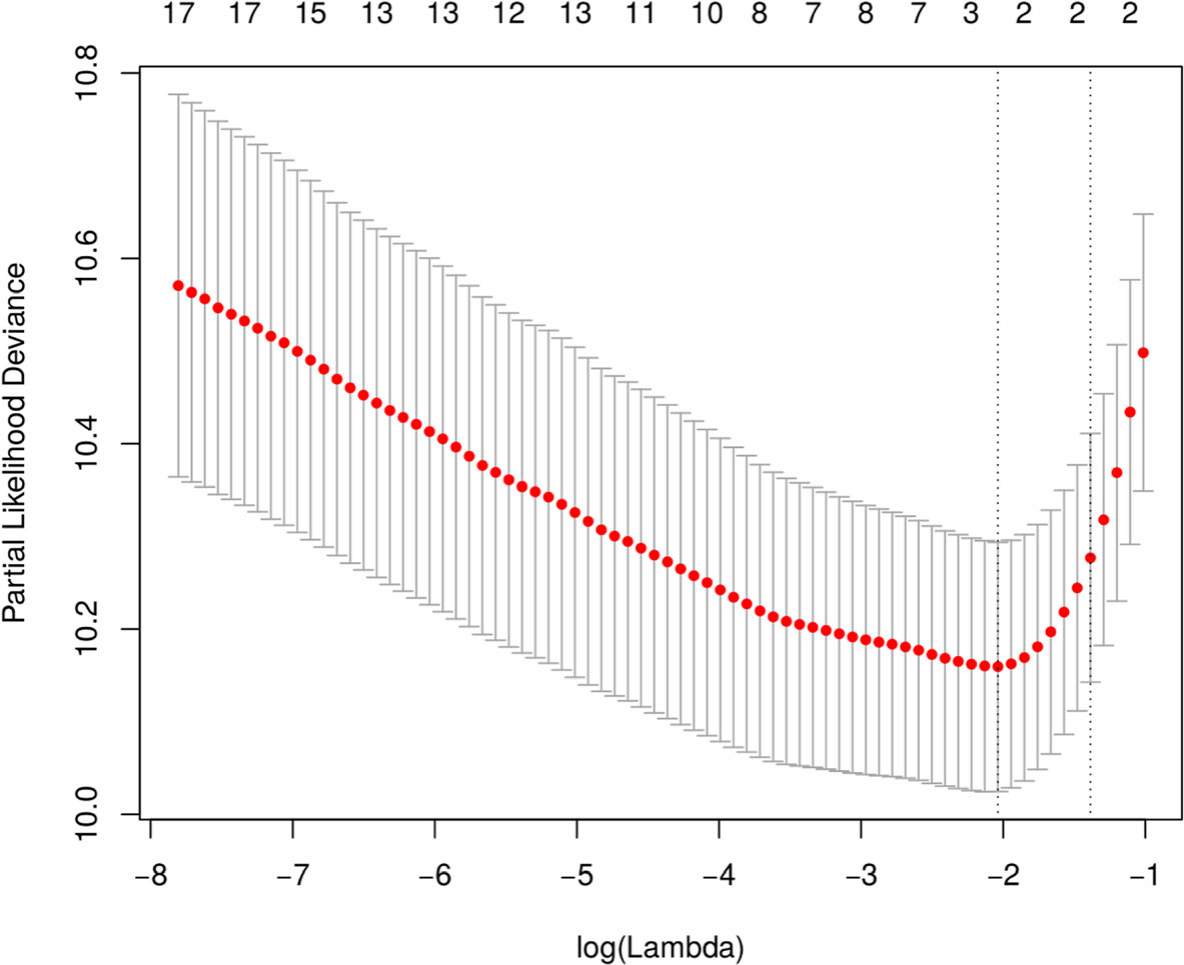
Elastic net regularization process with partial likelihood deviance plot. The minimum value corresponds to the covariates used for multivariate modeling (Cox model). The two vertical dot lines represent one standard deviation on each sides from the minimum value, corresponding to the chosen variables that better fit the model

**Fig. 4:**
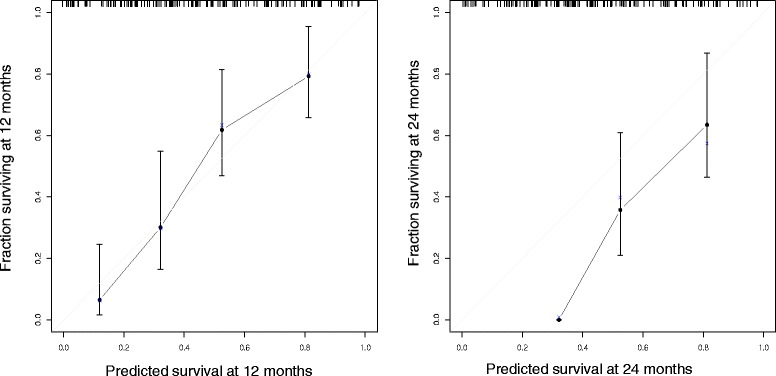
Calibration plot for Cox model. The predicted overall survival at 12 months shows good agreement among predicted and actual survival while the 24 months prediction shows lack of fit in the first group of patients and a general underestimate of the predicted survival

Using OS outcome for the analysis in the full dataset, the univariate log-rank test for covariates showed several significant results using all cases. Using this test, continuous numerical covariates were divided according to mobile threshold in order to better distinguish two categories of patients to fit the outcome. Table 5 summarizes the results of univariate log-rank test. Both clinical and radiomics covariates have been included and found significant. Figure 2 shows the cross-correlation matrix, indicating that there are multiple and complex cross-correlation among different covariates. This fact led to use a different approach for selecting the significant covariates that was the *elastic net regularization*. The result of such analysis showed that the most significant covariates with 1 standard deviation of partial likelihood deviance were the *compacity* and BCLC but Cox model fitting with stepwise regression returned only *compacity* as significant covariate (Cox model likelihood ratio test *p* < 0.001, *compacity p* < 0.0001, Fig. 3). AUC of ROC of the model is 0.8014 (C.I. = 0.7232–0.8797). The calibration plots of Cox model are shown in Fig. 4.

**Table 5:** Significant covariates with respect to the survival and related log-rank test *P*-Values

Covariate	*p*-Value	HR	95%CI
Total Dose	0.01	0.08	0.01–0.63
Localization of tumour	0.04	0.26	0.06–1.10
PV thrombosis	<0.001	3.51	2.01–6.13
AJCC Stage	0.001	1.42	1.10–3.01
BCLC Stage	<0.001	1.38	0.95–2.84
Child Class	0.04	1.63	1.02–2.61
AFP initial level	<0.001	0.39	0.25–0.63
Tumour volume	<0.001	0.25	0.16–0.40
Age	0.006	2.02	1.20–3.39
Entropy (Histogram)	0.02	1.70	1.09–2.67
Compacity	<0.001	0.22	0.14–0.36
Contrast	0.01	2.11	1.14–4.12
Entropy	0.04	1.61	1.03–2.88
Dissimilarity	0.01	2.17	1.14–4.12
HGRE	0.02	1.80	1.07–3.01
SRHGE	0.02	1.69	1.06–2.67
LRHGE	0.04	1.71	1.02–2.88
GLNU	<0.001	0.23	0.13–0.39
RLNU	<0.001	0.25	0.16–0.40
HGZE	0.02	1.83	1.09–3.07
SZHGE	0.01	1.80	1.12–2.87
GLNU	<0.001	0.25	0.15–0.39

Continuous numerical covariates have been split into two categories for calculating statistical test

## Discussion

The scope of our investigation was to perform a feasibility study to correlate some radiomics signatures to the clinical outcome in a retrospective analysis of a large cohort of patients already investigated and reported [29, 30]. As a matter of fact, texture analysis has been scarcely applied to primary liver cancer and, those studies, mostly focused on classification issues or on the development of decision aiding tools [23–26]. Some efforts have also been reported about the use of radiomics for the study of metastatic disease, particularly from colorectal cancer [27, 28].

In our study, the use of CT scan has given the chance to obtain images whose features have shown the possibility to be modeled according clinical and survival outcomes. The methodology implemented in this study is simple and easy to reproduce and the generation of the features was based on a validated package, freely available from the authors [31], all positive facts for a feasibility investigation; it is of course at the same time a limitation of the project not having introduced higher order texture analysis methods. Nevertheless, we hypothesized that, if a radiomics signature was to be found and possibly used in practice, and eventually shared,this should have been identified among the most robust and easy to implement categories.

The use of non-contrast enhanced treatment planning CT datasets, and the possibility to analyze the regions of interest identified as clinical target volume in the radiotherapy planning process, is another factor of interest of the study since enables an easy procedure and makes the process potentially available to all patients who will be scheduled for RT treatments. A limit of this approach, not appraised in our study is of course the sensitivity and robustness of the radiomics features to the segmentation process, the inter-observer variability (how different CTVs would be contoured by different radiation oncologists) and the possible presence of artifacts in the images (e.g. markers for positioning purposes). Apart from recognizing this limit, we shall consider that, unfortunately, this is a key problem for all kind of radiomics investigations. It is our opinion that predictive models will have to be built on large scale population datasets, from multiple institutions and from different scanning devices in order to encompass at maximum, the inherent variance of the input data. In this respect, our pilot study cannot of course solve the problem but, the further validation steps will try to appraise some of these points.

A second important factor to consider is the consistency of the patient’s cohort. In this study we focused on survival and on local control as a direct measure of the efficacy of the delivered treatment. A large cohort was available from an earlier retrospective study and it was used to compute all the radiomics features and to investigate the general aspect and OS. Unfortunately, the availability of clinical response outcome (LC) restricted the number of patients analyzed in the multivariate logistic regression models for that endpoint and this fact might led to get lower discrimination power models than the one achieved by analyzing the overall survival outcome. Concerning the whole dataset, despite the presence of a single significant covariate (*compacity*) the OS model is able to fit the outcome with a fair discrimination performance (AUC of ROC of the model 0.801). Looking at calibration plots the best survival estimate is given for the 12 months survival (Fig. 4) while the calibration at 24 months returned a lack of fit for the first group of patients and a general underestimate of survival prediction for the other two groups. This fact could be related to the lower median FUP time (16.6 months) respect to the length of this endpoint so longer time prediction could be achieved by increasing the FUP time and the number of observations.

An obvious limitation of this feasibility study, due to the issue mentioned above, is the lack of a validation based on an independent dataset. The limited consistency of the investigated cohort, prevented the possibility to separate it into training and testing subgroups and for this reason, a separate validation study is scheduled to be performed on a multicentric basis and with the (retrospective) inclusion of patients treated with either conventional or hypo-fractionated regimens. To provide a specific declination of this limitation, we might consider the fact that, since the full range of covariates did not return significant results, we applied thresholding methods to the covariates. The cut-off were identified based on the *p*-value analysis. Nevertheless, the absence of an external validation might question the robustness of these thresholds. This could in fact potentially cause a bias or a false positive because the explicit values might not be suitable for other population/situations. All this points to the necessity of a further set of investigations in this area, looking for an independent validation of the models.

## Conclusions

A radiomics signature made of a single textural feature allowed to fit a predictive model with a fair discrimination performance in HCC patients treated with volumetric modulated arc therapy. Further validation studies, at mono- and multi-centric level are mandatory and scheduled to confirm these findings.

## Abbreviations

2D: Two dimensional
AJCC: American joint committee on cancer
AUC: Area under the curve
BCLC: Barcelona Clinic Liver Cancer
CT: Computed tomography
CTV: Clinical target volume
FD: Full dataset
GLCM: Gray-level co-occurrence matrix
GLNU: Gray-Level Non-Uniformity for run
GLRLM: Grey level run length matrix
GLZLM: Grey level zone length matrix
HCC: Hepatocellular carcinoma
IMRT: Intensity modulated radiotherapy
LC: Local control
NGLDM: Neighborhood gray-level different matrix
OS: Overall survival
PTV: Planning target volume
RD: Restricted dataset
RECIST: Response evaluation criteria in solid tumors
RILD: Radiation induced liver disease
ROC: Receiver operating characteristic
SBRT: Stereotactic body radiation therapy
TACE: Trans-arterial chemo embolization
VMAT: Volumetric modulated arc therapy
